# Pediatric Arboviral Infections in Europe: Epidemiology, Clinical Features, Diagnosis and Prevention

**DOI:** 10.3390/v18090970

**Published:** 2026-09-03

**Authors:** Giulia Sturniolo, Silvia Garattini, Denis Doni, Marco Zacchello, Diletta Zanetti, Sofia Galzignato, Andrea Lo Vecchio, Vincenzo Baldo, Daniele Donà

**Affiliations:** 1 Division of Pediatric Infectious Diseases, Department of Women’s and Children’s Health, University of Padua, 35100 Padua, Italy; 2 Department of Women’s and Children’s Health, University of Padua, 35100 Padua, Italy; 3 Department of Translational Medical Sciences, University of Naples Federico II, 80131 Naples, Italy; 4 Department of Cardiac, Thoracic, Vascular Sciences, and Public Health, University of Padua, 35100 Padua, Italy; 5 Preventive Medicine and Risk Assessment Unit, Azienda Ospedale Università Padova, 35100 Padua, Italy

**Keywords:** arboviruses, pediatrics, Europe, West Nile virus, dengue virus, chikungunya virus, Zika virus, Oropouche virus, surveillance, One Health

## Abstract

Arboviral infections, including West Nile virus (WNV), dengue virus (DENV), chikungunya virus (CHIKV), Zika virus (ZIKV), and, to a lesser extent, Oropouche virus (OROV), pose a heterogeneous and evolving challenge for pediatric health in Europe. The increasing presence of competent vectors and international travel has resulted in both imported infections and, for selected arboviruses, documented autochthonous transmission events. Although pediatric cases are often underrecognized, children may develop severe manifestations, including neuroinvasive disease and congenital infection. Available data on pediatric arboviral infections in Europe remain limited and fragmented, complicating accurate assessment of disease burden and clinical risk. This review summarizes current evidence on the epidemiology, clinical presentation, diagnostic challenges, and prevention of selected arboviral infections in children within the European context, highlighting persistent gaps in pediatric-specific data and implications for preparedness and public health.

## 1. Introduction

Recent data from the European Centre for Disease Prevention and Control (ECDC) and recent systematic reviews indicate a progressive increase in arboviral circulation in Europe. Alongside the sustained circulation of West Nile virus (WNV), transmitted predominantly by *Culex* mosquitoes, *Aedes*-borne arboviruses such as dengue virus (DENV), chikungunya virus (CHIKV), and Zika virus (ZIKV) are emerging with increasing frequency [1]. Surveillance data show a growing number of both imported and autochthonous cases, with local transmission events reported annually in countries including France, Italy, and Spain, increasing by an estimated factor of 1.25 per year [1].

Delays in outbreak detection continue to limit the effectiveness of public health responses.

Climate change, increasing vector abundance, and global mobility are widely recognized as key drivers of this evolving epidemiological landscape [2,3].

Within this context, arboviruses of major relevance for pediatric health in Europe include West Nile virus (WNV), DENV, CHIKV, and ZIKV represent the most significant threats to human health, supported by the expanding distribution of competent vectors such as *Culex* mosquitoes, and *Aedes albopictus*. The presence and the spread of these vectors have facilitated local outbreaks in multiple European regions, suggesting that parts of Europe are transitioning toward increased arboviral endemicity [1,3,4,5].

Alongside these emerging infections, arboviruses already established in Europe continue to expand their range. Endemic viruses such as WNV, Toscana virus, and tick-borne encephalitis virus (TBEV) have shown increasing activity, with new transmission hotspots reported particularly in southeastern Europe and the Balkans [4,5]. Persistent circulation of WNV and Usutu virus has also been documented in countries including Croatia and the Netherlands, with ongoing zoonotic transmission and human neuroinvasive cases [4,5].

Although arboviral infections are more frequently reported in adults, pediatric cases present distinctive clinical and epidemiological challenges. Children may develop severe or atypical manifestations, including encephalitis, meningitis, or congenital disease, potentially related to age-specific physiological characteristics such as an immature blood–brain barrier and developing immune response [1,2,4,5,6,7].

In addition to these established threats, emerging arboviruses continue to attract attention. Oropouche virus (OROV), although currently endemic in Latin America, has recently been reported in imported cases in Europe. Experimental evidence suggests that *Aedes albopictus* could potentially act as a competent vector under favorable ecological conditions. While autochthonous pediatric OROV infections have not yet been reported in Europe, vertical transmission and neurological complications have been documented in endemic regions, highlighting the importance of surveillance and preparedness [8].

Despite increasing recognition of arboviral infections in Europe, the epidemiology, clinical presentation, and management of these infections in pediatric populations remain incompletely characterized [9,10,11].

## 2. Materials and Methods

To explore the epidemiology, clinical features, and surveillance of pediatric arboviral infections in Europe, this review focused on five arboviruses of major or emerging relevance in the European context: WNV, DENV, CHIKV, ZIKV, and OROV. These viruses were selected because of their documented or potential relevance for pediatric health in Europe, including established or expanding circulation, imported cases, documented autochthonous transmission, congenital or perinatal disease, neuroinvasive manifestations, or recent signals of public health concern.

A literature search was conducted in PubMed covering the period from January 2000 to January 2026, restricted to articles published in English. The search combined terms related to arboviruses, pediatrics, Europe, epidemiology, clinical manifestations, diagnosis, surveillance, prevention, and vaccines, together with virus-specific terms for WNV, DENV, CHIKV, ZIKV, and OROV. Additional epidemiological and surveillance data were retrieved from reports issued by international and regional public health agencies, including the European Centre for Disease Prevention and Control (ECDC) and national surveillance systems, as well as regulatory documents from the European Medicines Agency (EMA) and, when relevant to post-marketing safety data, the U.S. Food and Drug Administration (FDA).

Studies were included if they reported epidemiological, clinical, or surveillance data relevant to pediatric arboviral infections in Europe. Because pediatric-specific European data are limited for several arboviruses, studies from non-European endemic or epidemic settings were also considered when they provided clinically relevant age-specific information on pediatric disease presentation, severity, congenital or perinatal infection, diagnostic issues, outcomes, or prevention. These data were used to contextualize pediatric risk and preparedness in Europe, rather than to infer European disease burden directly. Studies focusing exclusively on adult populations, entomological research without human data, or experimental laboratory models were excluded, unless they provided essential European epidemiological, surveillance, vector, vaccine, or public health information relevant to the selected arboviruses. However, entomological and vector competence studies were considered when they directly informed the assessment of autochthonous transmission risk in Europe. Articles were also excluded if pediatric data could not be extracted or if infections were unrelated to the European context, unless they provided relevant comparative or pathophysiological insights.

Tick-borne arboviruses, including TBEV and CCHFV, Toscana virus, and Usutu virus were acknowledged as important components of the broader European arboviral landscape but were not included as full virus-specific sections, as this review focused on selected mosquito-borne and emerging arboviruses with recent or potential implications for pediatric preparedness in Europe. OROV was included despite its currently limited potential for autochthonous transmission in Europe because of its recent epidemiological expansion, and emerging evidence of vertical transmission and adverse fetal and neonatal outcomes.

Evidence was synthesized narratively, with particular attention to pediatric susceptibility, diagnostic challenges, and implications for surveillance and public health preparedness in Europe.

## 3. Results—Selected Arboviral Infections of Pediatric Relevance in Europe

Because pediatric data from Europe are scarce for several arboviruses, the following sections combine European epidemiological and surveillance evidence with pediatric clinical data from endemic or epidemic settings, when relevant to case recognition, clinical risk assessment, and preparedness in Europe. For each virus, evidence is organized around transmission and European epidemiology, pediatric clinical relevance, and key knowledge gaps.

### 3.1. West-Nile Virus

#### 3.1.1. Transmission and European Epidemiology

West Nile virus is discussed first because it is one of the most established arboviral threats in Europe and represents a major cause of neuroinvasive arboviral disease in temperate regions.

WNV is a mosquito-borne *Orthoflavivirus* maintained in an enzootic cycle between birds and mosquitoes of the genus *Culex*. Humans represent incidental, dead-end hosts within the transmission cycle. In Europe, *Culex pipiens* is the principal vector, and transmission occurs mainly during the summer and early autumn, typically peaking between July and September [12,13,14].

Cases of WNV transmission through blood transfusions and organ donations have been reported, including in children [15,16], but this risk of transmission has been significantly reduced by screening of blood and organ donors in areas of WNV activity. Although rare, congenital WNV infections (either transplacental or through breast milk) have been reported in children [17,18].

Over the past two decades, WNV transmission has progressively expanded northward and westward across Europe. Surveillance data indicate a substantial increase in both incidence and geographic distribution; as of early December 2025, a total of 1112 locally acquired human cases of WNV infection had been reported across 14 European countries, with 97 associated deaths in 2025. Most cases occurred in Italy, which alone accounted for 779 infections and 72 fatalities, representing the largest outbreak ever recorded in the country. Within Italy, the most affected areas included central and southern regions such as Lazio and Campania. Other countries reporting notable case numbers included Greece, France, Serbia, Romania and Spain, with smaller clusters observed across several additional nations [19,20]. Age-stratified data are collected within European WNV surveillance, but an aggregate number of pediatric cases is not provided in publicly available ECDC datasets. In Italy, children aged ≤ 14 years accounted for approximately 1% of the 366 WNND cases reported by mid-October 2025 [13].

A striking feature of the 2025 season was the geographic expansion of the virus: human cases have been reported for the first time in 35 regions across multiple countries, including Germany, Croatia, Kosovo, and Türkiye. This spread was mirrored by equid and bird outbreaks, with first animal detections also reported in Belgium and Netherlands, reflecting ongoing northward and westward expansion of the virus. These patterns are likely driven by a combination of climatic and ecological factors, such as expanding mosquito habitats and the role of migratory birds in viral dissemination [20].

Additionally, several countries—including Italy, France, Germany, Greece, Hungary, Croatia, and Spain—reported simultaneous human and animal cases, highlighting the importance of a One Health approach that brings together information from human, veterinary, and entomological surveillance to support early detection and monitoring of WNV circulation [20].

Overall, while the 2025 case count represents a 47% increase compared with the 10-year average, it remains below the peak observed in 2024. Nevertheless, the persistence of elevated transmission over three consecutive years suggests a transition toward a higher endemic level of WNV circulation in Europe, rather than a single anomalous outbreak [20].

These trends are widely attributed to environmental and climatic changes affecting mosquito ecology, including rising temperatures, altered precipitation patterns, and prolonged mosquito activity periods, which facilitate viral amplification and transmission [21].

Increasing transmission intensity may also result in a higher absolute number of pediatric infections, although most remain undiagnosed due to the predominantly asymptomatic or mild clinical presentation in this age group, which make them less likely to be detected by surveillance systems that primarily capture hospitalized or neuroinvasive cases [4,5,6].

For example, large surveillance datasets from the United States including more than 21,000 reported WNV cases between 1999 and 2007 identified only 1478 pediatric cases (<18 years), corresponding to approximately 4% of total reported infections [22]. However, seroprevalence studies during outbreaks suggest similar infection rates across age groups highlighting that the lower number of reported pediatric cases may reflect asymptomatic or undiagnosed infections rather than lower infection rates [23].

#### 3.1.2. Pediatric Clinical Relevance

Most WNV infections are asymptomatic or subclinical, accounting for approximately 70% of infections [24]. Among symptomatic pediatric patients, West Nile fever (WNF) is the most common presentation (about 67% of cases) and is characterized by fever, headache, rash, myalgia, and/or gastrointestinal symptoms. Rash is usually maculopapular, primarily affecting the chest, back, and upper limbs, generally resolves within one week, occurs in approximately 25–35% of symptomatic cases and has been associated with a more favorable clinical course [6,25,26].

Acute symptoms usually last between 3 and 10 days, although full recovery may take longer, with a median duration of around 60 days [27].

West Nile neuroinvasive disease (WNND) develops in about 1% of infections and typically manifests as meningitis, encephalitis, or acute flaccid paralysis (AFP). The proportion of WNND among total West Nile virus disease cases is similar in children and young adults (around 30%), but higher in elderly individuals (approximately 50%) [6,25].

In adults, advanced age and underlying chronic conditions are recognized risk factors for WNND [28]; however, in children, the determinants that modulate this risk remain incompletely defined.

Among reported pediatric cases, WNND represents a substantial proportion of diagnosed infections reflecting preferential recognition of severe clinical presentations [29].

In a large CDC surveillance analysis including 443 pediatric cases of WNV disease, approximately 40% presented with WNND, and among these, meningitis accounted for approximately 52% of cases, encephalitis for 39%, and AFP for 9% [22].

Unlike adults, in whom encephalitis is the predominant manifestation of WNND, pediatric patients more commonly present with meningitis [22]. Early symptoms may include fever, headache, vomiting, irritability and abdominal pain, while classical meningeal signs may be absent at disease onset [25,29].

Neurological involvement may evolve over several days and include seizures, altered mental status, tremor, movement disorders, or focal neurological deficits. Acute flaccid paralysis represents a rare but characteristic manifestation caused by anterior horn cell involvement and is characterized by rapidly progressive, often asymmetric weakness with preserved sensation [29].

Although rash is more common in WNF, it can also be observed, albeit less frequently, in patients with WNND, where it has been suggested as a potential marker of more severe disease and increased mortality [26].

Overall outcomes in pediatric patients are generally more favorable than in adults. Mortality among children with WNV infection is rare; in the CDC pediatric cohort described above, the case-fatality rate was approximately 1%, compared with around 14% among older adults [22]. This may reflect both the lower prevalence of underlying comorbidities and the lower incidence of encephalitis, which is associated with the poorest clinical outcomes.

Although long-term outcome data specifically in children are limited, available pediatric case series suggest that most patients recover completely, particularly after meningeal disease. However, residual neurological deficits—including motor weakness or cognitive difficulties—have been described following encephalitic presentations, with persistent neurological abnormalities reported in up to 86% of children with encephalitis [29,30,31].

Overall, WNND is usually monophasic, though recurrent weakness in AFP has been described [32]. Taken together, these pediatric findings highlight the importance of timely recognition of WNV infection during the transmission season, particularly in regions experiencing increasing viral circulation associated with environmental and climatic changes.

Pediatric-specific data on WNV infection in Europe remain limited. The true incidence of mild and asymptomatic infections in children is unknown, and age-specific risk factors for neuroinvasive disease are still poorly defined. Long-term neurological outcomes after pediatric WNND also remain insufficiently characterized. Strengthening pediatric surveillance and follow-up studies would improve estimates of disease burden and clinical risk.

### 3.2. Dengue

#### 3.2.1. Transmission and European Epidemiology

Dengue virus is primarily relevant to Europe through imported pediatric cases and, increasingly, through the risk of local transmission in areas where Aedes albopictus is established.

DENV is a mosquito-borne *Orthoflavivirus* transmitted by *Aedes* species. In Europe, *Aedes albopictus* represents the main competent vector. Transmission shows a marked seasonal pattern, with increased risk during the summer and early autumn when vector density is highest. The establishment of *Aedes albopictus*, together with increasingly favourable climatic conditions, has facilitated the potential for DENV transmission in temperate European regions [33,34].

Transmission through blood products is possible, especially during epidemics, and screening policies are in place in endemic areas [35]. Transmission through organ donations is anecdotal but several case reports are published [36]. Perinatal transmission, although rare, may also occur [37].

Over the past two decades, dengue epidemiology in Europe has progressively evolved from sporadic travel-associated infections to recurrent autochthonous transmission events in several EU/EEA countries. According to ECDC seasonal surveillance data, 304 autochthonous dengue cases were reported in Europe in 2024, representing the highest number ever documented and exceeding the combined total reported during the previous 15 years [33]. Autochthonous outbreaks have been documented in Croatia, France, Italy, and Spain, suggesting a progressive transition from sporadic transmission events toward recurrent seasonal circulation in parts of southern Europe [33,38].

This epidemiological shift has paralleled the progressive expansion of *Aedes albopictus* across Europe. A recent pan-European analysis showed that the number of affected regions increased from 129 regions across seven countries in 2010 to 358 regions in 14 countries by 2024 [33]. In parallel, the interval between the establishment of *Aedes albopictus* and the occurrence of autochthonous outbreaks progressively shortened from approximately 25 years in the 1990s to less than 5 years by 2024, while the interval between successive outbreaks decreased from approximately 12 years to less than 1 year over the same period [33].

France has reported the highest number of recurrent autochthonous dengue events in continental Europe, particularly in southern regions where *Aedes albopictus* is firmly established, while Italy has experienced increasingly large outbreaks over recent years. In 2020, Italy documented its first recognised autochthonous dengue outbreak with 11 locally acquired cases [39]. More recently, between August and October 2024, an outbreak caused by DENV serotype 2 in the city of Fano, Marche Region, resulted in 199 autochthonous cases, representing the largest dengue outbreak recorded in Italy to date [39].

Increasingly favourable climatic and ecological conditions are considered major drivers of the evolving epidemiological scenario of dengue in Europe. In Italy, the risk of local dengue transmission was found to peak between mid-August and the end of September, with transmission in central and southern regions potentially extending until mid-November because of persisting favourable temperatures [40]. Urban and peri-urban settings also appear particularly vulnerable to local transmission, with approximately 75% of affected European outbreak areas classified as urban or semi-urban regions [33].

National surveillance data from Italy further illustrate this changing epidemiological landscape. Although most dengue cases reported between 2015 and 2023 remained travel-related, Italy experienced its highest number of dengue notifications and local transmission chains in 2023, including the simultaneous circulation of multiple serotypes [41].

Pediatric involvement in autochthonous DENV transmission has been documented in several European countries, including Croatia, France, Spain, and Italy. However, age-specific data are not systematically reported for European outbreaks, preventing estimation of the overall proportion of locally acquired cases occurring in children. A recent systematic review identified pediatric cases within several European transmission events between 2010 and 2024, while confirming that pediatric case numbers were unavailable for several of the largest outbreaks [42]. In Italy, national surveillance data for 2025 reported DENV incidences of 0.46 and 1.60 cases per 1,000,000 population in the 0–9 and 10–19-year age groups, respectively [43].

Globally, children and adolescents bear a substantial proportion of the dengue burden, particularly in tropical and subtropical regions where transmission is endemic. A recent Global Burden of Disease analysis estimated more than 21.6 million dengue cases among individuals younger than 20 years in 2021, representing a 64% increase compared with 1990 [44].

Recent data from Latin America suggest a progressive epidemiological shift toward younger age groups. Historically, dengue in the Americas predominantly affected adults; however, countries such as Brazil and Colombia have reported a substantial increase in pediatric dengue cases and dengue-related fatalities since 2008. In Colombia, children younger than 15 years have accounted for approximately half of severe dengue cases since 2010 [45].

The substantial healthcare burden associated with pediatric dengue is further illustrated by large outbreak cohorts from endemic countries. During the 2023 outbreak in Bangladesh, 722 pediatric dengue hospitalizations and 17 deaths were reported in a single tertiary pediatric centre over a four-month period [46].

Despite the substantial burden observed in endemic settings, the true incidence of pediatric dengue is likely underestimated globally because many infections are asymptomatic or present as non-specific febrile illnesses, thereby reducing diagnostic recognition and surveillance capture outside outbreak contexts [44].

#### 3.2.2. Pediatric Clinical Relevance

Although severe pediatric dengue remains uncommon in Europe, global pediatric data are important for European clinicians because imported cases and autochthonous transmission events may expose children to a disease spectrum that is often unfamiliar in non-endemic settings.

Pediatric DENV infection may frequently remain clinically unrecognized. In a seroprevalence study including 400 asymptomatic children aged 1–12 years in Lahore, Pakistan, 25% showed evidence of previous DENV infection despite no prior clinical history of dengue, supporting the substantial burden of subclinical pediatric infections [47].

Among symptomatic pediatric cases, fever represents the most consistent clinical manifestation. In a prospective Nicaraguan cohort including 1405 laboratory-confirmed pediatric dengue cases collected over 18 years, fever and leukopenia were among the features most strongly associated with dengue compared with chikungunya and Zika infections [48]. However, the same study identified 62 laboratory-confirmed afebrile dengue cases, corresponding to 7.1% of all dengue cases, suggesting that reliance on fever-based case definitions alone may underestimate pediatric dengue burden [48]. Gastrointestinal manifestations are also frequent in children. In a prospective Bangladeshi cohort of 135 hospitalized pediatric dengue cases, abdominal pain was reported in 60.7% of patients, vomiting in 57%, and diarrhea in 17% [49]. In a Colombian cohort study from a hyperendemic region, children younger than 12 years more frequently presented with rash, pruritus, fever, hypotension, and thrombocytopenia compared with older patients [45].

Hematological abnormalities are common but non-specific. In the Bangladeshi pediatric cohort, thrombocytopenia was observed in 69.6% of cases, leukopenia in 47%, and elevated hematocrit in 36.3% [49]. Similar laboratory patterns have been consistently reported across pediatric dengue cohorts and may overlap with other acute febrile illnesses in childhood, particularly during the early stages of infection.

Pediatric dengue encompasses a broad spectrum of disease severity. Although most children develop uncomplicated dengue, clinically significant disease is not uncommon. In the same Bangladeshi hospital-based cohort, 16.3% of children fulfilled WHO criteria for dengue with warning signs and 15.6% were classified as severe dengue; however, these estimates should be interpreted in the context of a single-center cohort including only hospitalized children [49]. Severe pediatric dengue does not necessarily present with overt hemorrhagic manifestations. Plasma leakage, pleural effusion, ascites, and hemodynamic instability may occur during the critical phase of illness and represent major determinants of clinical deterioration in children [49]. In pediatric cohorts from endemic settings, younger age and secondary DENV infection have been associated with increased risk of severe disease [50]. In a large retrospective pediatric study from Nicaragua, severe dengue occurred predominantly in infants aged 4–9 months and in children aged 5–9 years, while secondary dengue infection was associated with increased disease severity [51]. This age distribution is consistent with the well-described phenomenon of severe primary dengue occurring as maternally derived anti-DENV antibodies decline to sub-neutralizing levels, facilitating antibody-dependent enhancement during infancy [52].

Although severe pediatric dengue remains relatively uncommon in Europe, imported pediatric cases may require hospitalization and, occasionally, intensive care support. Increasing international travel and the progressive establishment of autochthonous transmission in Europe therefore underscore the importance of maintaining diagnostic awareness of dengue in febrile children, particularly in those with recent travel history or epidemiological exposure [53].

In Europe, pediatric dengue data remain scarce and are mostly derived from imported cases or outbreak reports with limited age-specific detail. Important gaps include the incidence and clinical spectrum of autochthonous pediatric dengue, hospitalization rates, warning signs in European children, and the impact of previous flavivirus exposure or vaccination on disease severity. These gaps are increasingly relevant as local transmission events become more frequent in southern Europe.

### 3.3. Chikungunya

#### 3.3.1. Transmission and European Epidemiology

CHIKV is an arthropod-borne alphavirus transmitted by *Aedes* mosquitoes, predominantly *Aedes albopictus* and *Aedes aegypti* [35,37]. The widespread establishment of *Aedes albopictus* in Europe has created favourable conditions for local transmission in temperate regions [54,55]. Vertical transmission from viremic mothers to neonates during the peripartum period has been well documented, as well as transmission through transfusion of blood products collected during donor viremia [54,56].

Originally isolated in Tanzania and historically associated with sporadic outbreaks in Africa and Asia, CHIKV expanded globally after 2005, causing large-scale epidemics in the Indian Ocean region, the Americas, and parts of Asia [54,55]. Three main genotypes are recognized: West African, East/Central/South African (ECSA), and Asian [54].

In Europe, autochthonous CHIKV transmission has been documented since 2007, when Italy reported the first recognized outbreak in continental Europe, involving 217 laboratory-confirmed locally acquired cases in the Emilia-Romagna region following viral introduction by a traveller returning from India [55]. Subsequent outbreaks confirmed the establishment of local transmission potential in areas colonized by *Aedes albopictus*. According to a retrospective epidemiological analysis covering 2007–2023, a total of 4730 chikungunya cases were reported across 22 European countries, with most cases occurring in the United Kingdom (1038 cases, 21.9%), France (929, 19.6%), Germany (684, 14.5%), and Italy (644, 13.6%) [57]. Notably, Italy also experienced a large outbreak in 2017, accounting for 270 reported cases [1]. Seasonal surveillance conducted by the ECDC indicates persistent risk of CHIKV circulation during the vector season in EU/EEA countries where *Aedes albopictus* is established [58].

In 2025, Europe experienced the largest chikungunya season recorded to date, with nearly 800 locally acquired cases reported in France and more than 380 in Italy, alongside multiple transmission clusters documented across several affected regions, with extensive autochthonous transmission and an unprecedented geographic expansion into areas previously considered unsuitable for sustained transmission [58,59]. Recent analyses further suggest that the frequency of local transmission events in Europe has progressively increased over time, paralleling the geographic expansion of competent vectors and increasingly favourable climatic conditions [1,59].

Pediatric cases have been documented during autochthonous CHIKV outbreaks in both Italy and France, although age-specific reporting has been inconsistent and does not allow the pediatric contribution to European outbreaks to be reliably quantified [42]. In Italy, national surveillance data for 2025 reported CHIKV incidences of 1.16 and 3.56 cases per 1,000,000 population in the 0–9 and 10–19-year age groups, respectively [43].

Beyond Europe, chikungunya represents a substantial pediatric health burden globally. A systematic review and meta-analysis including 142 pediatric studies found that CHIKV infection remains significantly underrecognized and underreported in children, with the highest prevalence estimates observed during epidemic periods [60].

Recent longitudinal cohort data from Brazil showed that laboratory-confirmed CHIKV infection occurred in 16.3% of children and adolescents followed prospectively over a three-year period, supporting the substantial circulation of CHIKV in pediatric populations living in endemic areas [61].

#### 3.3.2. Pediatric Clinical Relevance

The clinical presentation of chikungunya in children differs from that observed in adults and varies substantially with age, with a broader spectrum of dermatologic and neurologic manifestations and less prominent musculoskeletal involvement than in adults [56,62]. The reported frequency of asymptomatic infection varies considerably across outbreak settings and study designs. A review by Ritz et al. reported asymptomatic infection in approximately 35–40% of pediatric cases, although this proportion is considerably lower in neonates and young infants [62]. In a recent prospective Brazilian cohort including 348 children and adolescents, 53 laboratory-confirmed CHIKV infections were identified, of which only 9.4% were completely asymptomatic, while 83.0% were polysymptomatic and 7.5% oligosymptomatic [61].

A global systematic review and meta-analysis including 142 pediatric studies identified fever, rash, and arthralgia as the most frequently reported clinical manifestations, with thrombocytopenia representing the most common laboratory abnormality [60]. Clinical manifestations vary across cohorts but consistently identify fever and rash as the predominant presenting features. In a Indian cohort of 58 hospitalized children, in which fever was present in 94.8% of patients, skin rash in 55.2%, vomiting in 43.1%, myalgia in 39.7%, and arthralgia in only 13.8%, further supporting that joint symptoms are less prominent in children than in adults [63]. These findings are consistent with observations from the 2005–2006 Réunion outbreak, where a retrospective cohort of 86 children identified fever in virtually all cases, maculopapular rash in approximately 55%, arthralgia in about 48%, and myalgia in 27% of patients [64].

Cutaneous manifestations include erythematous maculopapular eruptions, hyperpigmentation, desquamation, and bullous lesions, while neurologic symptoms such as altered sensorium, seizures, and meningoencephalitis have been consistently reported in pediatric cohorts [56,62].

Severe disease occurs predominantly in younger children, particularly infants younger than one year [60]. In the same Réunion Island cohort, 25 of 86 children (30%) presented with neuropsychiatric involvement, including seizures (52%), confusion (32%), behavioural disturbances (32%), meningeal syndrome (16%), and coma in the most severe cases; two children died because of neurological complications [64]. In an Indian hospital-based cohort of 58 children, severe complications included capillary leak syndrome (46.6%), thrombocytopenia (44.8%), acute kidney injury (19.0%), shock (10.3%), encephalopathy (10.3%), meningitis (10.3%), and acute respiratory distress syndrome (8.6%), with an overall mortality of 3.4% (2/58 patients) [63].

Although chronic rheumatic sequelae are less common than in adults, they are not negligible in pediatric populations. In the Brazilian prospective cohort, chronic arthralgia persisting for more than three months was observed in 12% of RT-PCR-confirmed cases, while the ESPID review estimated that persistent arthralgia or arthritis may affect 5–11% of children up to two years after infection [62].

Neonates represent a particularly vulnerable population. Vertical transmission occurs predominantly in the setting of maternal peripartum viremia and may approach 50% when maternal infection occurs around delivery [62]. A recent Brazilian case series by Ferreira et al. reported an even higher transmission rate of 62% (18/29) among mothers who were viremic near delivery, with only one asymptomatic infected neonate, whereas all other vertically infected infants required admission to the neonatal intensive care unit [65]. Clinical manifestations typically develop between the third and seventh day of life and include fever, rash, edema, thrombocytopenia, and lymphopenia, while severe cases may develop intracerebral hemorrhage, status epilepticus, encephalopathy, or multiorgan failure requiring intensive care support [56,62]. In the Brazilian series, neurological manifestations—including hypoactivity, irritability, weak sucking, seizures, or encephalitis—were observed in 70% of infected neonates, followed by fever (55%), cutaneous manifestations (50%), respiratory involvement (45%), and hematological abnormalities (40%) [65]. Long-term neurological sequelae remain a major concern. A recent systematic review and meta-analysis by Zhou et al. found that maternal CHIKV infection during pregnancy was associated with an increased risk of adverse neurodevelopmental outcomes in offspring (pooled RR 1.87, 95% CI 1.30–2.70), supporting the need for prolonged developmental follow-up of exposed infants [66].

For chikungunya, European pediatric data remain limited, particularly regarding neonatal infection, hospitalization, neurological complications, and chronic musculoskeletal outcomes. Further studies are needed to define age-specific disease burden during European outbreaks and to guide follow-up of infected infants and children, especially after perinatal or early-life infection.

### 3.4. Zika

#### 3.4.1. Transmission and European Epidemiology

In Europe, Zika virus remains mainly a travel-associated infection, but it retains major pediatric relevance because of congenital infection and the need for pregnancy-related counselling, diagnosis, and follow-up.

ZIKV is a mosquito-borne *Orthoflavivirus* primarily transmitted by *Aedes aegypti* and *Aedes albopictus* [67]. Sexual transmission has been clearly demonstrated, with infectious virus and viral RNA detected in semen and spermatozoa for prolonged periods after acute infection [68]. Viral persistence has also been documented in vaginal secretions and whole blood, supporting the potential for sexual transmission from both male and female individuals beyond the acute phase [69].

Vertical transmission from mother to fetus can also occur at any stage of pregnancy, as ZIKV is able to cross the placental barrier and viral RNA has been detected in placental tissue and amniotic fluid, confirming in utero fetal exposure [70,71]. The marked tropism of ZIKV for placental and neural cells underlies both congenital infection and neurodevelopmental injury [71].

ZIKV circulation was historically confined to Africa and Asia; however, large outbreaks occurred in the Americas between 2015 and 2018, leading to global recognition of its neurotropic and teratogenic potential [67]. Following these epidemics, transmission declined but has not been eliminated, with ongoing circulation and sporadic outbreaks reported in multiple regions worldwide [67]; as per the most recent WHO epidemiological review, Africa and the Americas and south-east Asia appear most involved [72]. Because a substantial proportion of ZIKV infections are asymptomatic or mildly symptomatic, the true extent of transmission is likely underestimated [67]; a recent review estimated a global seroprevalence of ZIKV up to 21%, mainly coming from the Americas and South-East asia following the aforementioned epidemics [73].

In Europe, ZIKV infection has been reported almost exclusively as an imported disease associated with travel to endemic regions. Despite the presence of competent *Aedes* vectors in several European countries, sustained autochthonous transmission has not been documented, and the overall epidemiological risk remains low [67]. Migration represents another possible point of entry for the disease; however, data do not seem to support a significant transmission risk; for example an Italian study screened the serum samples of 64 migrants coming into the country in the first six months of 2023 from sub-Saharan Africa and South-East Asia; only a single patient presented positive for anti-ZIKV IgM; however, no detectable RNA levels or positive anti-ZIKV IgG were found either in that patients or in the other screened individuals [74].

#### 3.4.2. Pediatric and Congenital Relevance

The pediatric relevance of ZIKV is dominated by congenital infection, whereas postnatal infection in children is usually mild but may rarely be associated with neurological complications. Congenital ZIKV infection results from vertical transmission during pregnancy and may occur following both symptomatic and asymptomatic maternal infection. Multiple lines of epidemiologic, clinical, and laboratory evidence support a causal relationship between prenatal ZIKV infection and congenital brain abnormalities, including microcephaly and other severe neurologic defects; for instance, during the 2015 outbreak in Brazil, 12 out of 72 pregnant women who presented positive for ZIKV-RNA showed fetal ultrasound abnormalities; accordingly, in 2013–2014 French Polynesia it was estimated that 1% of neonates of mother infected with ZIKV in the first trimester had microcephaly, a 50 times increase compared to the baseline prevalence [70]. Congenital Zika Syndrome (CZS) encompasses a spectrum of abnormalities, including microcephaly with partial collapse of the skull, intracranial calcifications, ventriculomegaly, cortical malformations, craniofacial disproportion, ocular lesions, sensorineural hearing loss, hypertonia, seizures, arthrogryposis, and impaired postnatal neurodevelopment [75]. Long-term outcomes are often severe, with increased mortality and substantial neurodevelopmental disability reported among affected children [70].

Postnatal ZIKV infection in children and adolescents accounts for between 10 and 30% of total ZIKV postnatal cases, depending on the country and period [76,77]; clinical presentation is usually asymptomatic or mild, similar to adults. A 2020 English systematic review found that after an incubation period of approximately 3–14 days, symptomatic cases typically presented with low-grade fever (55–99% of cases), pruritic maculopapular rash (29–100%), non-purulent conjunctivitis (11–64%), arthralgia (14–48%), myalgia (6–57%) and headache (21–50%) [75]. Neurologic complications represented the major indicator of severe disease; although they were confirmed by the same review to be rare (at around 1% of cases in the pediatric population), they still have been described, particularly during large outbreaks such as during the 2015–2016 epidemics in Brazil. These include Guillain–Barré syndrome, meningoencephalitis, myelitis, and acute disseminated encephalomyelitis [75].

Another potential influencing factor may be coinfection, mainly with other arboviruses (e.g., DENV, CHIKV) in tropical areas [48]. ZIKV disease in the context of coinfections remains unexplored, however a recent review found that such coinfections could reach up to 50% of cases in areas such as Brazil during outbreaks [78]; from the available data the clinical impact compared to ZIKV monoinfections do not appear to be significant, although further studies appear to be required [78].

The main pediatric gaps for ZIKV in Europe concern imported infections during pregnancy, timely access to diagnosis, interpretation of serology in populations with previous flavivirus exposure or vaccination, and long-term follow-up of exposed infants. Although sustained autochthonous transmission has not been documented in Europe, surveillance remains important in areas where competent Aedes vectors are established.

### 3.5. Oropouche

#### 3.5.1. Transmission and Relevance for Europe

Oropouche virus is included as an emerging arbovirus to monitor rather than as an established European threat. Its current relevance for Europe is mainly related to imported cases, diagnostic awareness in returning travellers, and emerging concerns about vertical transmission.

OROV is an orthobunyavirus transmitted to humans primarily through the bite of infected *Culicoides paraensis* biting midges, which represent the principal vector responsible for urban outbreaks in endemic areas of Central and South America [79,80]. Transmission occurs through interconnected sylvatic and urban cycles involving wild vertebrate hosts and human–vector–human spread, respectively. Although other arthropods have been investigated as potential vectors, current evidence indicates that *C. paraensis* is the main vector sustaining urban transmission [80].

In Europe, the principal urban vector *Culicoides paraensis* has not been reported, and experimental studies have demonstrated very low or absent vector competence of locally established mosquito species, including *Aedes albopictus* and *Culex pipiens*, substantially limiting the current risk of sustained local transmission [80,81,82]. Vertical transmission following maternal infection has been documented and represents an emerging route of clinical relevance [83].

First identified in 1955 in Trinidad and Tobago, OROV has become endemic in large parts of the Amazon Basin and other regions of Central and South America where it has caused recurrent outbreaks over several decades [79]. A marked resurgence has been observed during the 2023–2025 period, characterized by a sharp increase in reported cases, and geographic expansion beyond historically endemic areas [79,80]. According to the Pan American Health Organization, 16,239 laboratory-confirmed cases and four deaths were reported across the Americas in 2024, with confirmed transmission documented in Brazil, Bolivia, Peru, Colombia, Cuba, Ecuador, Guyana, Panama, and Barbados, representing the largest OROV outbreak recorded to date [84].

In Europe, OROV infection remains rare and has been identified exclusively in travelers returning from endemic areas. In 2024, 19 imported cases were reported in EU/EEA countries including 12 cases in Spain, 5 in Italy, and 2 in Germany, almost all linked to recent travel to Cuba and one to Brazil, all without evidence of onward local transmission [81]. To date, no autochthonous transmission has been documented in Europe. The absence of the main competent vector and the low vector competence of locally established mosquitoes support the assessment that the current risk of sustained OROV transmission in Europe remains low, although continued surveillance is warranted [80,81,82].

To date, no dedicated pediatric surveillance studies or population-based cohorts have systematically evaluated the incidence, clinical burden, or outcomes of OROV infection in children. Most available evidence derives from mixed-age outbreak investigations or reports focusing on congenital infection, precluding robust conclusions regarding the epidemiology of postnatally acquired disease in pediatric populations [79,83]. Consequently, the true burden of OROV infection in children remains unknown and represents an important knowledge gap, highlighting the need for prospective pediatric epidemiological studies.

Evidence regarding congenital OROV infection remains limited but is rapidly evolving. To date, no population-based studies have quantified the incidence of vertical transmission or its attributable fetal risk. Available evidence derives mainly from mixed-age outbreak investigations or reports focusing on congenital infection, including a Brazilian study that identified OROV IgM in 6 of 68 samples from newborns with microcephaly of previously unknown etiology, supporting the possibility of congenital infection while highlighting the need for prospective studies to define its true epidemiological burden [83,85].

#### 3.5.2. Pediatric and Congenital Relevance

Oropouche virus infection most commonly presents as an acute febrile illness. A systematic review and meta-analysis by Wang et al., including 15 studies and 806 patients, identified fever (100%) and headache (95%) as the predominant clinical manifestations, followed by myalgia (72%), arthralgia (58%), retro-orbital pain (56%), and nausea or vomiting (44%), whereas rash was reported in only 11% of cases [86].

The clinical presentation is largely non-specific and overlaps with that of other arboviral infections, contributing to diagnostic challenges in endemic and epidemic settings.

Neurological involvement, although uncommon, represents the most severe manifestation of OROV infection. Central nervous system involvement has been reported in approximately 4% of cases and includes aseptic meningitis, meningoencephalitis, and, more rarely, Guillain–Barré syndrome, supporting the neuroinvasive potential of the virus [10,87].

Data on postnatally acquired OROV infection in children are extremely limited. To date, no pediatric-specific clinical series are available that allow a distinct characterization of disease presentation in children compared with adults. Consequently, descriptions of postnatal OROV infection in pediatric populations rely on general clinical patterns observed across mixed-age cohorts, and age-specific differences in clinical course or severity cannot be reliably defined. Nevertheless, isolated pediatric cases have been reported, including a laboratory-confirmed infection in a Haitian child presenting with an acute febrile illness [88].

By contrast, increasing evidence indicates that OROV infection during pregnancy may result in severe fetal and perinatal outcomes. A Brazilian case series identified evidence of OROV-specific IgM in cerebrospinal fluid and/or serum in 6 of 68 newborns with microcephaly of previously unknown etiology, while one fatal case also had OROV RNA detected in cerebrospinal fluid and multiple tissues, providing stronger evidence of congenital infection [85]. Additional reports have described miscarriage, stillbirth, neonatal death, and congenital central nervous system abnormalities, including microcephaly, ventriculomegaly, cerebral atrophy, corpus callosum dysgenesis, posterior fossa abnormalities, and arthrogryposis, with detection of viral RNA in placental and fetal tissues providing biological evidence for vertical transmission [11,83]. Detection of OROV RNA in semen has also been reported, suggesting a theoretical potential for sexual transmission; however, confirmed sexually transmitted cases have not been documented to date [83].

For OROV, the main gaps include the absence of pediatric-specific clinical cohorts, uncertain incidence of vertical transmission, incomplete characterization of congenital outcomes, and limited diagnostic availability outside endemic settings. In Europe, the absence of the principal vector and the low competence of locally established mosquitoes suggest a low current risk of sustained transmission, but imported cases require clinical and public health awareness.

**Figure 1:**
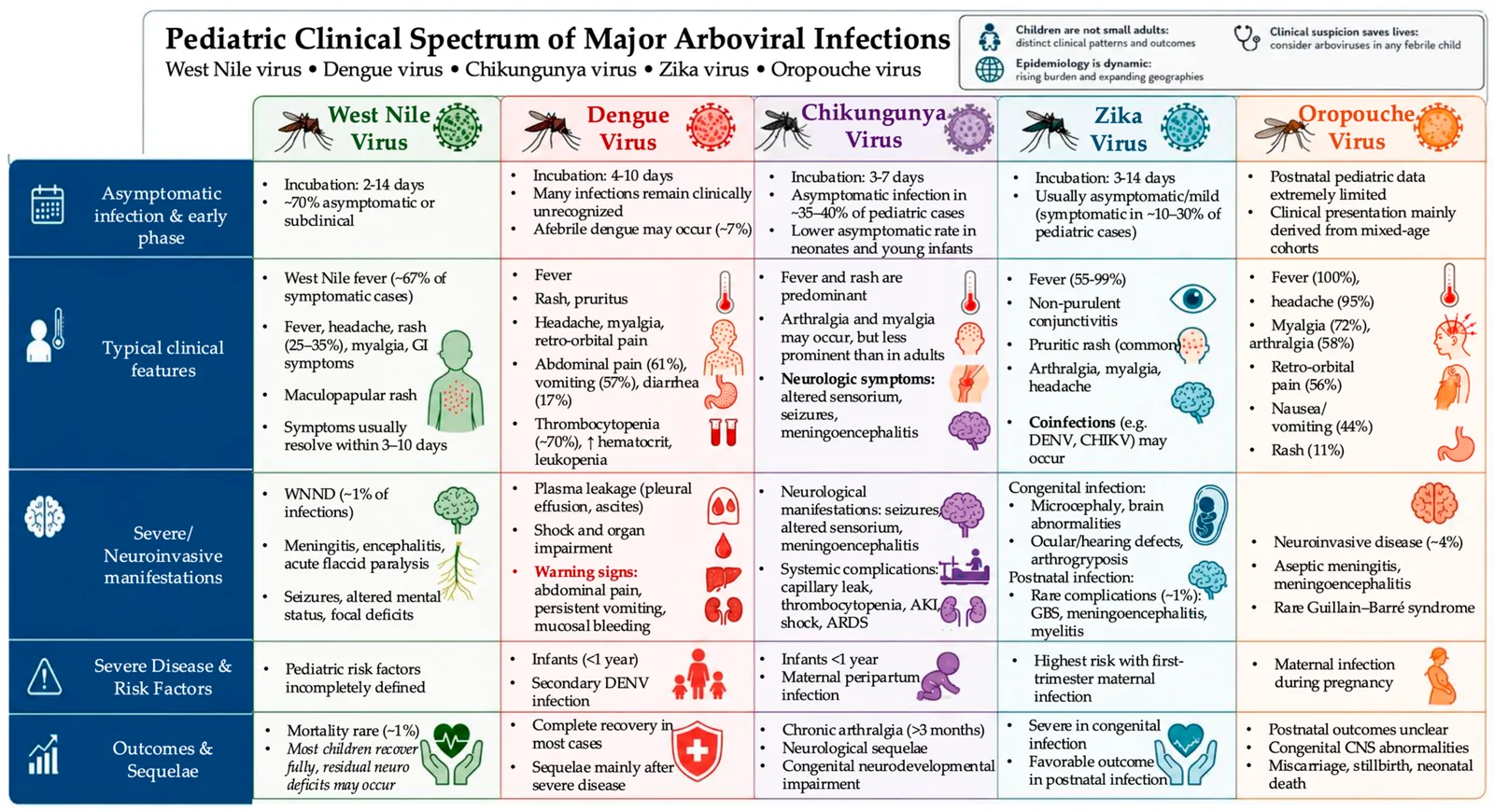
Major arboviral infections relevant to pediatric health in Europe. (Generative AI tools (ChatGPT, GPT-5.1 (OpenAI)) were used during the conceptual development of the graphical layout of this figure, AI tools were not used to generate the figure text. The scientific content and structure of the figure were provided by the authors, and the instruction given to the tool was: “Create a graphical representation of the supplied scientific content and structure.”).

A comparative summary of the selected arboviruses, including their relevance for pediatric health in Europe, clinical spectrum, risk factors for severe disease, and outcomes, is provided in Figure 1.

### 3.6. Diagnosis

In pediatric patients, the diagnosis of arboviral infections, including WNV, DENV, CHIKV, ZIKV, and OROV, relies primarily on the recognition of a compatible clinical syndrome within an appropriate epidemiological context. Arboviral infections in children frequently present with overlapping and non-specific manifestations, such as acute febrile illness, rash, arthralgia or myalgia, conjunctivitis, gastrointestinal symptoms, and, less commonly, neurological involvement, making clinical features alone insufficient for etiological differentiation [2,89].

Laboratory confirmation is strongly influenced by the timing of specimen collection relative to symptom onset. During the early acute phase, molecular detection by real-time reverse transcription polymerase chain reaction (RT-PCR) constitutes the most informative diagnostic approach for DENV, CHIKV, ZIKV, and OROV, whereas its sensitivity for WNV is limited due to low and transient viremia [90,91]. WNV RNA may also be detected in urine, where it can persist longer than in blood, extending the window for molecular detection [91,92].

For WNV, serologic testing therefore is commonly used for diagnosis, with detection of virus-specific IgM antibodies in serum or cerebrospinal fluid supporting recent infection, particularly in cases of suspected neuroinvasive disease [90].

As viremia declines, serologic testing becomes central for all arboviruses. Detection of virus-specific IgM antibodies supports recent infection and may be performed on serum and, when clinically indicated, on cerebrospinal fluid [93,94]. Interpretation of serologic results requires particular caution in pediatric patients because of substantial cross-reactivity among orthoflaviviruses, including WNV, DENV, and ZIKV. In this context, plaque-reduction neutralization testing (PRNT) is used to confirm the specificity of serological results, particularly for flaviviruses, although these assays are not available in all diagnostic laboratories and may require referral to reference laboratories [94,95,96].

For DENV, diagnostic approaches commonly combine molecular testing or NS1 antigen detection in the acute phase with IgM serology in later stages of illness, while IgG serology is primarily used to document previous exposure rather than acute infection [90]. For CHIKV and ZIKV, IgM antibodies typically become detectable after the first week of illness, whereas IgG antibodies mainly reflect past infection and are not reliable markers of acute disease [56,94]. For OROV, available evidence indicates that molecular detection and IgM serology represent the principal diagnostic tools, while IgG testing has limited utility for acute diagnosis in endemic settings [80].

Ancillary laboratory abnormalities, such as leukopenia, thrombocytopenia, atypical lymphocytes, or elevated transaminases, may support clinical suspicion, particularly in dengue, but remain nonspecific and cannot establish a diagnosis independently [89]. Imaging studies have no role in routine diagnosis and should be reserved for selected cases with complications, such as ascites and fluid effusion, suspected neuroinvasive disease or persistent inflammatory arthropathy, as they do not contribute to etiologic confirmation [97].

During pregnancy, additional prenatal diagnostic evaluation may be warranted for maternal arboviral infections with potential fetal involvement. For ZIKV, molecular detection by RT-PCR on amniotic fluid is considered the most specific means for prenatal diagnosis of intrauterine infection, and detailed ultrasound with neurosonography is essential for detecting features such as microcephaly, ventriculomegaly, or brain calcifications in the fetus [98,99]. Maternal–fetal transmission of ZIKV has been documented throughout pregnancy, with viral RNA detected in fetal losses and infants with congenital abnormalities, although the precise predictive value of prenatal diagnostic tests remains incompletely defined [70]. A practical summary of the diagnostic approach to pediatric arboviral infections according to the phase of illness is provided in Table 1.

### 3.7. Treatment and Prevention

For the arboviral infections discussed in this review, no virus-specific antiviral therapy has demonstrated proven clinical efficacy, and clinical management is therefore supportive.

Treatment is based on adequate hydration, maintenance of hemodynamic stability, and symptomatic relief of fever and pain.

During the acute phase, the use of non-steroidal anti-inflammatory drugs and salicylates should be avoided when DENV infection cannot be confidently excluded, because of the associated risk of hemorrhagic complications [100].

Hospitalization and close clinical monitoring are recommended for patients with severe disease, neurological involvement, poor oral intake, respiratory compromise, relevant comorbidities, or very young age, as well as for neonates born to mothers with peripartum arboviral infection, particularly chikungunya or ZIKV infection [56,93]. In selected cases, post-acute or long-term sequelae require specialized follow-up. Persistent inflammatory musculoskeletal manifestations following CHIKV infection may warrant rheumatologic evaluation and treatment with conventional disease-modifying anti-rheumatic drugs, along with physiotherapy to improve function and reduce disability [97,101].

In congenital ZIKV infection, management remains supportive but requires long-term, multidisciplinary follow-up to address neurological, ophthalmologic, and developmental outcomes [93].

In the absence of broadly available vaccines for most arboviruses, prevention relies primarily on non-pharmacological measures, including vector control and personal protective behaviors to reduce exposure to mosquito bites. This approach remains particularly relevant in non-endemic settings, where population immunity is low and prevention strategies are essential to limit transmission [102].

### 3.8. Vaccines

Despite the increasing public health relevance of arboviral infections, vaccine availability remains limited and heterogeneous across different viruses, with particularly important implications for pediatric populations. Overall, vaccine development for arboviruses has progressed unevenly, reflecting challenges related to viral diversity, incomplete understanding of immunopathogenesis, fluctuating epidemiology, and safety considerations in vulnerable groups such as children and pregnant women [103,104,105].

Among the arboviruses discussed in this review, DENV is currently the only one for which vaccines are licensed with potential applicability in children. In the European Union, a live-attenuated tetravalent dengue vaccine developed by Takeda (TAK-003; commercial name Qdenga^®^) has been approved for use in individuals from 4 years of age, regardless of prior dengue exposure, and is administered as two doses given three months apart. This authorization is supported by phase 3 clinical trial data in children and adolescents aged 4–16 years, showing significant efficacy against virologically confirmed dengue and a marked reduction in dengue-related hospitalizations, with an acceptable short-term safety profile [106,107].

By contrast, the first licensed dengue vaccine, CYD-TDV (Dengvaxia^®^), is no longer authorized/marketed in the EU; its prior restriction to seropositive individuals reflected post-hoc analyses indicating an increased risk of severe dengue in seronegative recipients, including pediatric populations [108,109]. This phenomenon has been interpreted in the context of antibody-dependent enhancement (ADE), whereby non-neutralizing or subneutralizing antibodies may facilitate viral entry during subsequent DENV infections and contribute to severe disease manifestations [110].

For CHIKV, vaccine development has advanced substantially. In the European Union, both a live-attenuated vaccine (Ixchiq^®^) and a recombinant virus-like particle (VLP) vaccine (VIMKUNYA^®^; CHIKV VLP) are authorized for use in individuals aged 12 years and older [111,112].

In addition, a Phase 3 clinical trial of the VLP vaccine has been initiated in children aged 2–11 years, aiming to extend pediatric indications [113]. Phase 2 pediatric data for Ixchiq^®^ reported acceptable short-term safety and persistence of neutralizing antibodies up to 12 months in children aged 1–11 years [114]. However, post-marketing safety concerns have been reported for Ixchiq^®^ in the United States, where the FDA suspended the biologics license in August 2025 [115]. These findings underscore that data on long-term protection, real-world effectiveness, and pediatric safety remain limited and that continued pharmacovigilance is essential [54].

In contrast, no licensed vaccines are currently available for WNV, ZIKV, or OROV, and prevention remains centered on vector control, personal protection, and surveillance [102].

Table 2 summarizes the currently licensed and late-stage vaccine candidates relevant to pediatric populations.

## 4. Discussion

The progressive expansion of arboviral transmission in Europe highlights the need for enhanced surveillance that includes pediatric populations. Despite the increasing number of reported arboviral outbreaks, children remain underrepresented in surveillance systems, leading to an incomplete understanding of disease dynamics, age-specific risk, clinical presentation, long-term outcomes, and burden. This review also highlights that arboviral infections should not be regarded as a single, homogeneous public health challenge. Rather, each virus currently poses a distinct epidemiological and clinical scenario in Europe, with different implications for pediatric surveillance, diagnosis, and preparedness [1,4,5].

Pediatric surveillance is particularly important for WNV, DENV, CHIKV, and ZIKV, although the priorities differ according to the epidemiology of each infection. WNV, already established in several European regions, requires continued strengthening of integrated surveillance systems, with systematic inclusion of pediatric data within existing One Health frameworks, as pediatric neuroinvasive disease remains uncommon but potentially severe [4,5]. By contrast, the expansion of *Aedes albopictus* and the increasing frequency of autochthonous DENV and CHIKV outbreaks indicate that preparedness should now focus on ensuring that pediatric healthcare systems can rapidly recognize, diagnose, and respond to locally acquired infections rather than considering these viruses exclusively in returning travelers [1,3,4,5]. ZIKV remains primarily relevant because of imported maternal infection and congenital disease, whereas OROV does not currently represent an established European threat. For both viruses, preparedness should therefore focus on establishing predefined diagnostic and referral pathways for suspected maternal, congenital, and imported infections, while ensuring that clinicians working in obstetric, neonatal, and pediatric settings remain able to recognize these conditions promptly [2,6].

It is important to note that the reviewed arboviruses do not represent a homogeneous threat to children. While WNV is primarily associated with neuroinvasive disease, DENV remains the arbovirus responsible for the greatest pediatric disease burden worldwide, and CHIKV may cause severe disease in neonates and young infants, including neurological complications following perinatal transmission [2,6]. ZIKV differs from the other arboviruses because of its well-established teratogenic potential, whereas evidence regarding OROV remains limited but is rapidly evolving particularly with respect to vertical transmission and congenital infection [70,83,85].

In children, arboviral infections are frequently mild or asymptomatic, but when symptomatic they may present with fever, rash, arthralgia, or neurological involvement. These nonspecific features overlap with other common pediatric illnesses, leading to underdiagnosis and underreporting [2,6]. From a clinical perspective, arboviral infections should be considered in children with compatible symptoms during the vector season, after travel to endemic or outbreak areas, or in the context of local transmission alerts. This is particularly relevant for children presenting with unexplained fever and rash, meningoencephalitis, acute flaccid weakness, neonatal illness after maternal peripartum infection, or congenital abnormalities following maternal exposure during pregnancy. The implementation of standardized, age-specific diagnostic criteria and laboratory protocols, such as those proposed in the MERMAIDS-ARBO study, could substantially improve case detection and outbreak response across Europe [7]. Diagnostic preparedness is essential because the performance and interpretation of laboratory tests depend on the timing of sample collection, the phase of illness, and the potential for serologic cross-reactivity among flaviviruses. Improved access to molecular testing, serology, and confirmatory neutralization assays would support more accurate case classification, especially in children with neurological manifestations or suspected congenital infection. Beyond postnatally acquired infections, several arboviruses raise important concerns during pregnancy and the perinatal period. Congenital ZIKV infection remains the best-characterized example, but increasing evidence also supports vertical transmission of CHIKV and OROV, with reports of adverse fetal, neonatal, and neurodevelopmental outcomes [65,66,83]. These observations suggest that maternal, fetal, and pediatric health should be considered together when assessing the impact of emerging arboviruses and designing surveillance strategies. Accordingly, arboviral preparedness should include obstetric surveillance, neonatal evaluation, and long-term developmental follow-up when maternal infection occurs during pregnancy or around delivery.

Prevention remains central in Europe, where population immunity is generally low and autochthonous transmission is still focal and seasonal. Vector control, personal protective measures, blood and organ safety, travel medicine, and targeted risk communication are therefore essential components of preparedness. Vaccine availability is evolving but remains uneven across arboviruses. Dengue and chikungunya vaccines may create new opportunities for prevention, while also raising questions regarding pediatric indications, safety, target populations, and implementation in non-endemic or intermittently affected European settings.

Enhanced pediatric surveillance would not only clarify the burden of infection but also serve as an early indicator of local transmission. Children’s social and behavioral patterns, including outdoor activity and close contact networks, make them potential sentinel populations in emerging foci. Pediatric surveillance should extend beyond simple case reporting: systematic collection of age-specific clinical presentations, neurological and congenital manifestations, and long-term outcomes would provide information that cannot be inferred from adult surveillance alone and is essential for planning pediatric preparedness strategies. Integration of pediatric data into national and regional arboviral monitoring frameworks would strengthen early warning capacity and guide resource allocation for prevention and control. This integration should ideally occur within a One Health framework, combining pediatric, adult, obstetric, veterinary, entomological, and environmental data to identify transmission signals early and to coordinate public health responses. Rather than expanding surveillance uniformly across all arboviruses, future pediatric surveillance should be tailored to the principal public health priorities of each infection. This includes strengthening the integration of pediatric data within established WNV surveillance systems, improving recognition and characterization of pediatric DENV and CHIKV infections during autochthonous outbreaks, and establishing standardized maternal–infant surveillance pathways for ZIKV and OROV.

In summary, Europe’s arboviral landscape is characterized by the growing importance of WNV, DENV, CHIKV, and ZIKV, and, to a much lesser extent, by potential risks associated with OROV. The inclusion of pediatric populations in surveillance systems is essential to close current knowledge gaps, improve diagnostic accuracy, and enable timely public health interventions. A coordinated One Health approach, combining human, veterinary, and entomological data, remains fundamental to effectively address the evolving epidemiology of arboviruses in Europe. Future research should prioritize pediatric-specific surveillance, age-stratified clinical presentation and outcomes, long-term follow-up after neuroinvasive or congenital infection, and the evaluation of vaccine strategies relevant to children and adolescents.

## 5. Limitations

This review has several limitations. First, available evidence on pediatric arboviral infections in Europe remains fragmented and largely derived from surveillance reports or small observational studies. Second, heterogeneity in national surveillance systems and diagnostic criteria may contribute to underestimation of pediatric cases. Third, restricting the search to English-language publications may have led to the exclusion of relevant local reports from endemic regions. Finally, because pediatric European data are scarce for several arboviruses, this review also considered evidence from endemic or epidemic non-European settings when clinically relevant. These data help contextualize pediatric risk and case recognition but cannot be used to directly estimate the burden of disease in European children.

## 6. Conclusions

In conclusion, arboviral infections represent an evolving challenge for pediatric health in Europe, characterized by heterogeneous epidemiology and variable clinical presentations. Although severe pediatric disease and congenital infections remain relatively uncommon, children are affected during both imported and autochthonous transmission events and may contribute to the early detection of local circulation. However, pediatric-specific epidemiological and clinical data remain limited, particularly for emerging arboviruses. Strengthening pediatric surveillance, improving diagnostic capacity, and integrating pediatric data into existing monitoring frameworks will be essential to enhance Europe’s preparedness for future arboviral emergence.

## Figures and Tables

**Table 1:** General diagnostic approach to pediatric arboviral infections.

Phase of Illness	Main Diagnostic Tools	Notes
Early acute phase (first week after symptoms onset)	RT-PCR on blood ± urine/CSF	Limited sensitivity for West Nile virus due to low and transient viremia (more persistent in urine)
Later acute/early convalescent phase (from the end of the first week after symptoms onset)	IgM serology (serum ± CSF)	IgM may become detectable from approximately day 5, depending on the virus; sensitivity generally increases after the first week CSF testing relevant in suspected neuroinvasive disease
Positive flavivirus IgM	PRNT confirmation	Required due to cross-reactivity (WNV, DENV, ZIKV)
Supportive findings	CBC, liver enzymes	Nonspecific; supportive only
Imaging	Selected complicated cases	Not diagnostic

**Table 2:** Licensed and late-stage vaccines for selected arboviruses with relevance to pediatric populations.

Virus	Vaccine (Type)	Regulatory Status in the EU	Pediatric Age Indication	Key Notes
**Dengue virus**	TAK-003 (Qdenga^®^), live-attenuated tetravalent	Authorized	≥4 years	Two-dose schedule (3-month interval); phase 3 trials in children/adolescents showed efficacy against virologically confirmed dengue and reduced hospitalizations
**Dengue virus**	CYD-TDV (Dengvaxia^®^), live-attenuated tetravalent	No longer authorized/ marketed	—	Prior restriction to seropositive individuals; increased risk of severe dengue in seronegative recipients, including children
**Chikungunya virus**	Ixchiq^®^, live-attenuated	Authorized	≥12 years	Phase 2 pediatric data (1–11 years) showed short-term safety and antibody persistence; post-marketing safety concerns reported in the US
**Chikungunya virus**	VIMKUNYA^®^ (CHIKV VLP), recombinant VLP	Authorized	≥12 years	Phase 3 trial ongoing in children aged 2–11 years to extend pediatric indications
**West Nile** **virus**	—	Not available	—	No licensed vaccines
**Zika virus**	—	Not available	—	No licensed vaccines
**Oropouche** **virus**	—	Not available	—	No licensed vaccines

## Data Availability

No new data were created or analyzed in this study. Data sharing is not applicable to this article.

## Abbreviations

The following abbreviations are used in this manuscript:

ADE	Antibody-dependent enhancement
AFP	Acute flaccid paralysis
CBC	Complete blood count
CDC	Centers for Disease Control and Prevention
CHIKV	Chikungunya virus
CSF	Cerebrospinal fluid
CZS	Congenital Zika syndrome
DENV	Dengue virus
ECDC	European Centre for Disease Prevention and Control
ECSA	East/Central/South African
EEA	European Economic Area
EFSA	European Food Safety Authority
EMA	European Medicines Agency
EU	European Union
FDA	U.S. Food and Drug Administration
OROV	Oropouche virus
PRNT	Plaque reduction neutralization test
RNA	Ribonucleic acid
RT-PCR	Reverse transcription polymerase chain reaction
TBEV	Tick-borne encephalitis virus
US	United States
VLP	Virus-like particle
WHO	World Health Organization
WNE	West Nile encephalitis
WNF	West Nile fever
WNND	West Nile neuroinvasive disease
WNV	West Nile virus
ZIKV	Zika virus
